# Changes in GABAergic markers accompany degradation of neuronal function in the primary visual cortex of senescent rats

**DOI:** 10.1038/s41598-017-15006-3

**Published:** 2017-11-02

**Authors:** Yanxia Ding, Yuan Zheng, Tao Liu, Ting Chen, Changhua Wang, Qiushuang Sun, Mutian Hua, Tianmiao Hua

**Affiliations:** 1 College of Life Sciences, Anhui Normal University, Wuhu, Anhui 241000 China; 2 Department of Materials Science and Engineering, University of California, Los Angeles, CA 90095 USA

## Abstract

Numerous studies have reported age-dependent degradation of neuronal function in the visual cortex and have attributed this functional decline to weakened intracortical inhibition, especially GABAergic inhibition. However, whether this type of functional decline is linked to compromised GABAergic inhibition has not been fully confirmed. Here, we compared the neuronal response properties and markers of GABAergic inhibition in the primary visual cortex (V1) of young adult and senescent rats. Compared with those of young adult rats, old rats’ V1 neurons exhibited significantly increased visually evoked responses and spontaneous activity, a decreased signal-to-noise ratio and reduced response selectivity for the stimulus orientation and motion direction. Additionally, the ratio of GABA-positive neurons to total cortical neurons in old rats was significantly decreased compared with that in young rats. Expression of the key GABA-synthesizing enzyme GAD67 was significantly lower in old rats than in young rats, although GAD65 expression showed a marginal difference between the two age groups. Further, expression of an important GABA_A_ receptor subunit, GABA_A_R α_1_, was significantly attenuated in old rats relative to young ones. These results demonstrate that ageing may result in decreased GABAergic inhibition in the visual cortex and that this decrease in GABAergic inhibition accompanies neuronal function degradation.

## Introduction

Visual ability deteriorates during the normal ageing process^1–3^. Many types of age-related visual impairment, such as a reduction of orientation/motion-direction discrimination and contrast detection, are chiefly attributed to the decline of visual cortical neuron function^4^. Indeed, numerous previous studies have reported age-dependent functional degradation of cortical neurons across hierarchical sensory processing stages in several mammalian species and have attributed these decreases in neuronal function to compromised intracortical inhibition, especially GABAergic inhibition^4–12^.

A few recent studies examined age-related changes in GABAergic markers in various brain regions of several mammalian species. These studies reported a decreased proportion of γ-aminobutyric acid (GABA)-positive neurons^13–15^ as well as modified expression of GABA-synthesizing enzymes^16–18^ and GABA receptors during the ageing process^18–21^. However, the results of these studies are inconsistent and, in some cases are even contradictory^16–19,21,22^. Therefore, it is difficult to reach a convincing conclusion on how GABAergic inhibition changes during ageing. Furthermore, although changes in GABAergic markers are observed, it is unknown whether these changes parallel the functional degradation of cortical neurons that occurs during ageing.

In this study, we examined and compared the response properties of neurons in visual cortical area V1 in old and young adult rats using *in vivo* single-unit recording technique. After recording, brain tissue containing the V1 region was recovered, and the levels of intracortical GABAergic markers were assessed by immunofluorescent labelling and western blotting. We measured the proportion of GABA-positive neurons and expression of the GABA-synthesizing enzymes GAD65 and GAD67 as well as expression of the important GABA_A_ receptor subunit α_1_. The aim of this study was to determine whether the degradation of neuronal function in cortical area V1 that occurs during senescence is accompanied by a reduction of GABAergic inhibition in the V1 region.

## Results

### Age-related functional changes in V1 neurons

To determine whether V1 cortical neurons of old rats exhibit functional degradation relative to V1 neurons of young adult rats, we examined the neuronal responses of V1 neurons to visual stimuli with varied orientations and motion directions. A total of 79 neurons in 6 young adult rats and 83 neurons in 6 old rats were studied. The distribution of cell number across the depth corresponding to different cortical layers (layers I, II–III, IV, V and VI)^23^ in the two age groups showed no significant difference (Chi-square test, p = 0.721). Additionally, among the cells studied, the proportion of simple cells to complex cells in old rats (29/54) was not significantly different from that in young adult rats (32/47) (Chi-square test, p = 0.465).

**Figure 1:**
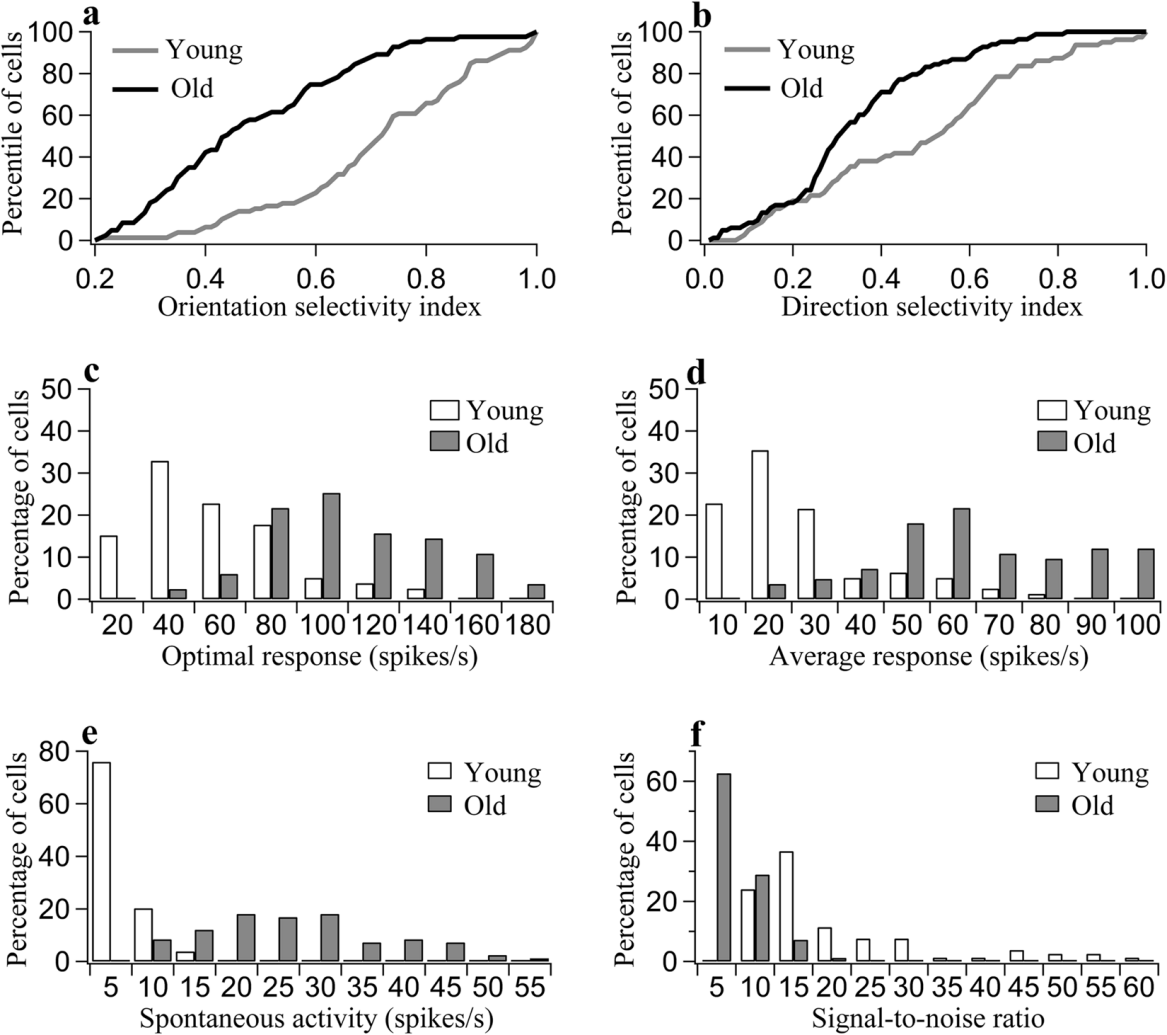
Statistics showing the response properties of V1 neurons in young adult and old rats. (**a**,**b**) Percentile of neurons as a function of the orientation selectivity index (**a**) and motion direction selectivity index (**b**) in young adult and old rats. (**c**,**d**) Percentage of neurons with different ranges of optimal response to the optimal stimulus (**c**) and different ranges of average response to all stimuli (**d**) in young and old rats. (**e**) Percentage of neurons with different ranges of spontaneous activity in young and old rats. (**f**) Percentage of neurons with different ranges of signal-to-noise ratio in young and old rats.

The response selectivity of V1 neurons to stimuli with different orientations and directions of motion was compared in the two groups of rats. In old rats, the orientation selectivity indexes (OSIs) of the neurons were distributed as follows: 19.3% of the cells had OSI ≥ 0.65; in 38.5% of the cells, 0.4 < OSI < 0.65; and 42.2% of the cells had OSI ≤ 0.4. These values differed significantly from the OSI values found in young adult rats; in the young animals, 68.4% of the cells had OSI ≥ 0.65, in 26.5% of the cells, 0.4 < OSI < 0.65, and 5.1% of the cells had OSI ≤ 0.4 (Chi-square test, p < 0.0001) (Fig. 1a). Student’s t-test showed that the mean OSI value in the group of old rats (0.48 ± 0.18) was significantly lower than the mean OSI value in the group of young rats (0.71 ± 0.18) (two-tailed t-test, p < 0.000001). Similarly, in old rats, neurons with different motion direction selectivity index (DSI) values were distributed as follows: 12% of the cells had DSI ≥ 0.6; in 16.9% of the cells, 0.4 < DSI < 0.6; and 71.1% of the cells had DSI ≤ 0.4, whereas in young adult rats, 38% of the cells had DSI ≥ 0.6, in 22.8% of the cells, 0.4 < DSI < 0.6, and 39.2% of the cells had DSI ≤ 0.4 (Chi-square test, p < 0.0001) (Fig. 1b). The average DSI in old rats (0.33 ± 0.17) was also significantly smaller than that in young adult rats (0.48 ± 0.25) (two-tailed t-test, p = 0.000029). These results show that V1 cortical neurons of old rats exhibit a significant decrease in response selectivity for stimulus orientation and direction of motion compared with V1 cortical neurons of young adult rats.

To further clarify whether the decreased response selectivity of V1 neurons in old rats resulted from a decreased response to the optimal stimulus orientation, increased response to non-optimal orientations or both, we compared the optimal responses (ORs) of neurons to their preferred stimuli as well as the average response (AR) of neurons to all stimulus orientations in young adult and old rats.

The majority of neurons (70.9%) in young adult rats had OR values less than or equal to 60 spikes/s, whereas most neurons (91.4%) in old rats had OR values greater than 60 spikes/s (Fig. 1c). The t-test showed that the mean OR value in the old group (99.1 ± 29.5) was significantly higher than that in the young adult group (47.1 ± 27.9) (two-tailed t-test, p < 0.000001). Thus, the mean OR in the old group was increased by 110.4% relative to the young group. Additionally, the majority of neurons (79.7%) in young adult rats exhibited AR values lower than 30 spikes/s, whereas 91.5% of the neurons in old rats displayed AR values higher than 30 spikes/s (Fig. 1d). The mean AR in the old group (61.5 ± 23.2) was also significantly larger than the mean AR in the young adult group (21.9 ± 17.3) (two-tailed t-test, p < 0.000001). Thus, the mean AR in the old group was increased by 180.8% relative to the young group. These results show that although the visually evoked response of V1 neurons to stimuli of all orientations increased significantly during ageing, the response to stimuli with non-optimal orientations increased more than the response to stimuli with optimal orientations.

We also compared the spontaneous activity (SA) of V1 neurons in the two age groups. The majority of neurons (75.9%) in young adult rats had SA below 5 spikes/s, whereas most neurons (91.6%) in old rats had SA above 10 spikes/s (Fig. 1e). The mean SA of V1 neurons in the old group (24.7 ± 11.7) was significantly larger than the mean SA in the young adult group (3.4 ± 2.6) (two-tailed t-test, p < 0.000001). The mean SA of V1 neurons in the group of old rats increased by 626% relative to the mean SA of V1 neurons in the group of young rats.

Due to the large increase in spontaneous neuronal activity, the mean signal-to-noise ratio (SNR) of V1 neurons in old rats (5.2 ± 3.2) was significantly lower than that in young adult rats (18.3 ± 14.7) (two-tailed t-test, p < 0.000001). The majority of neurons (75.9%) in young rats had SNR values larger than 10, whereas most neurons in old rats (91.6%) had SNR values smaller than 10 (Fig. 1f). Relative to young rats, the mean SNR in old rats was reduced by 71.6%.

Taken together, the above analyses demonstrate that V1 neurons in old rats do exhibit functional degradation in response to visual stimuli. This finding is consistent with previous reports in different mammalian species^4–7^.

To explore whether the age-related decrease in the function of V1 cortical neurons was coupled to a change in GABAergic inhibition, we examined GABAergic markers in the V1 region, including the proportion of GABA-positive neurons as well as the expression of GABA-synthesizing enzymes and ionotropic GABA_A_ receptors.

### The proportion of GABA-positive neurons

**Figure 2:**
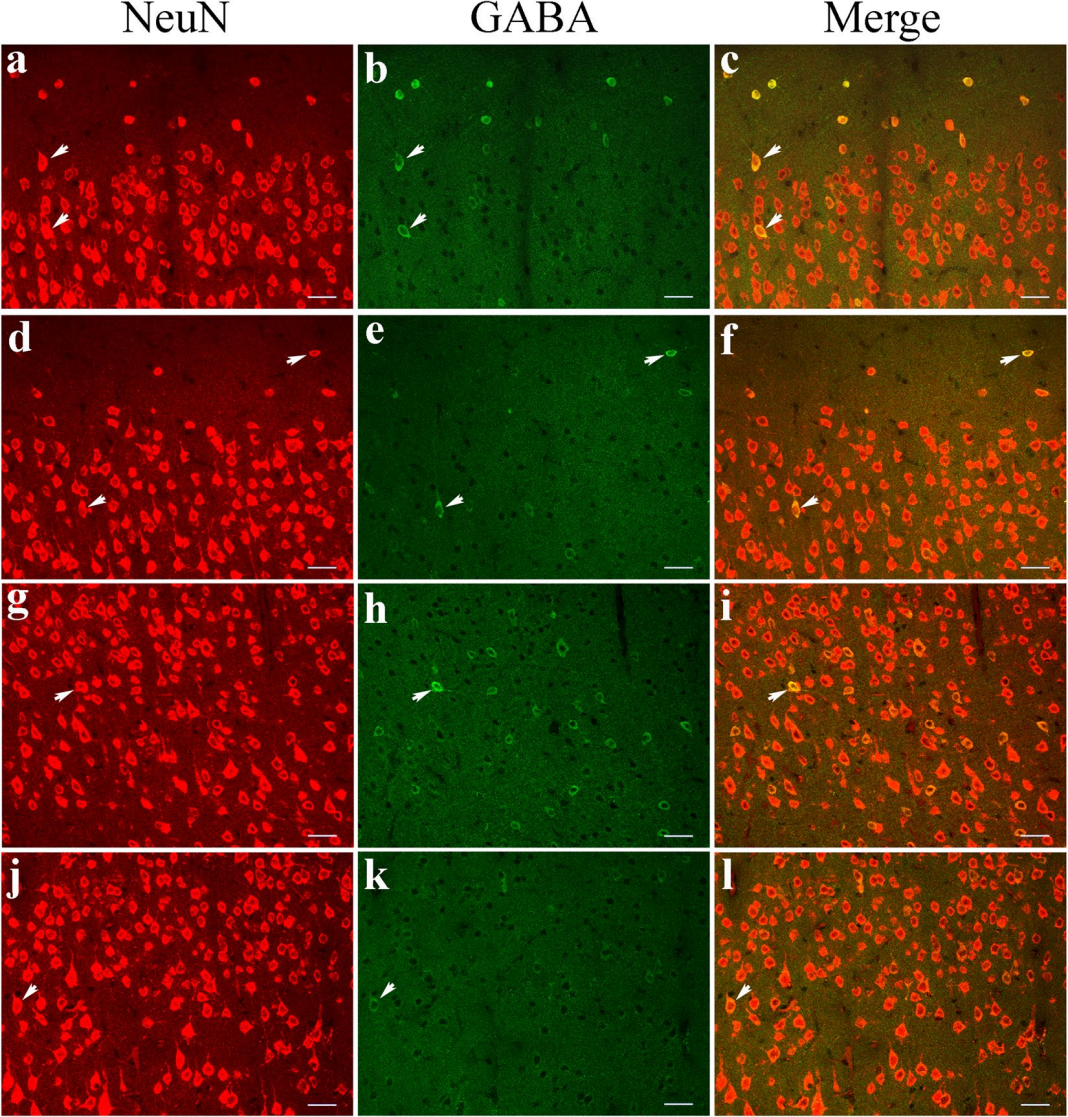
Immunofluorescence double labelling of total cortical neurons (NeuN-positive neurons) and GABAergic neurons (GABA-positive neurons) in different cortical layers in the V1 cortex of young adult (**a**–**c**,**g**–**i**) and old rats (**d**–**f**,**j**–**l**). **a**–**f** and **g**–**l** represent double-labelled samples from cortical layers I–III and IV–VI, respectively. Arrows  indicate typical double-labelled neurons. The scale bar equals 30 μm.

To examine the proportion of GABA-positive neurons in the V1 cortical region, we double-labelled cortical neurons in sections from this region with fluorescent antibodies to NeuN and GABA. NeuN fluorescence was observed in the cell bodies of all cortical neurons, and the distribution of NeuN-positive neurons within each cortical layer was comparable in young adult and old rats (Fig. 2a,d,g, and j). GABA fluorescence was quite evident within the cell bodies of GABAergic neurons and was also visible in the main dendritic arbors and axonal terminals. The distribution of GABA-positive neurons and double-labelled neurons in all cortical layers in old rats was limited compared to that observed in young adult rats (Fig. 2b,c,e,f,h,i,k, and l). Although the mean density of total neurons (NeuN-positive neurons) within each cortical layer in the V1 region of old rats was not significantly different from that of young adult rats (two-way ANOVA, F(1, 600) = 1.196, p = 0.275), the mean density of GABA-positive neurons in the V1 region of old rats was significantly lower in all cortical layers than in young adult rats (two-way ANOVA, F(1, 600) = 1635.711, p < 0.0001) (Table 1). Furthermore, the mean ratio of GABA-positive neurons to total neurons in each cortical layer in old rats was also significantly reduced compared to that in young adult animals (two-way ANOVA, F(1, 600) = 1491.297, p < 0.0001) (Table 1). Relative to young adult rats, the ratio of GABA-positive neurons to total neurons in layers I, II–III, IV, V and VI of old rats decreased by 64.8%, 64.2%, 69.6%, 61.9% and 71.3%, respectively.

**Table 1:** The mean density of total neurons and GABA-positive neurons and ratios of GABA-positive neurons to total neurons in various cortical layers (I, II–III, IV, V and VI) in the primary visual cortex of young adult and old rats.

Subjects	Cortical layer				
	I	II–III	IV	V	VI
Total cortical neurons (cells/mm^2^)					
Young rats	3309 ± 817	14,668 ± 2948	17,882 ± 2928	16,261 ± 3413	13,098 ± 3551
Old rats	3282 ± 522	14,568 ± 2749	17,401 ± 1879	15,868 ± 2275	12,887 ± 3869
GABA-positive neurons (cells/mm^2^)					
Young rats	2765 ± 839	2139 ± 542	3034 ± 681	2779 ± 495	1821 ± 534
Old rats	954 ± 502	771 ± 242	881 ± 196	1117 ± 426	483 ± 151
Proportion of GABA-positive neurons (%)					
Young rats	82.6 ± 10.6	14.8 ± 3.0	16.8 ± 2.1	18.1 ± 5.6	14.3 ± 3.2
Old rats	29.1 ± 13.8	5.3 ± 1.3	5.1 ± 1.3	6.9 ± 2.3	4.1 ± 1.9

### Expression of GAD65 and GAD67

**Figure 3:**
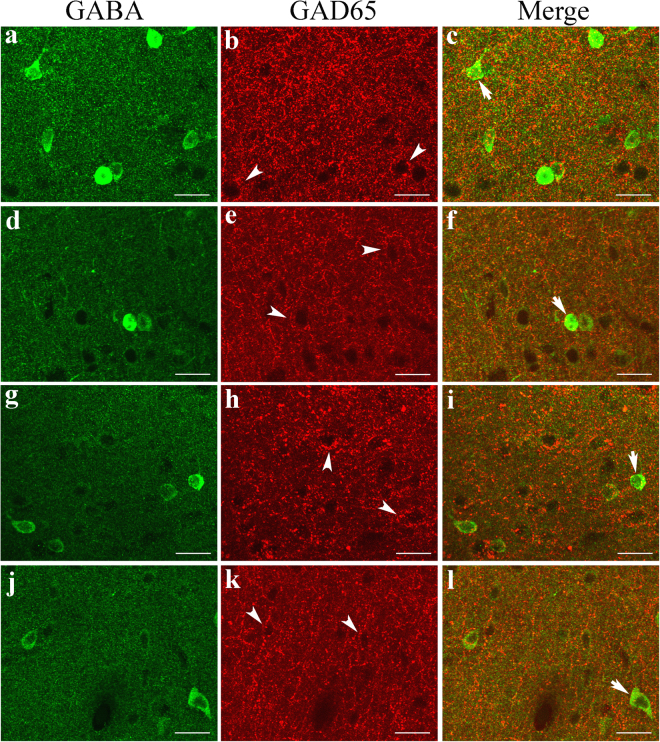
Fluorescence double labelling of GABA and the GABA-synthesizing enzyme GAD65 in different cortical layers in the V1 cortex of young adult (**a**–**c**,**g**–**i**) and old rats (**d**–**f**,**j**–**l**). **a**–**f** and **g**–**l** represent double-labelled samples from cortical layers I–III and IV–VI, respectively. Arrows  indicate neurons that show weak double labelling of GABA and GAD65, and arrowheads  indicate GAD65 fluorescence around non-GABAergic neurons (black hole). The scale bar equals 20 μm.

**Figure 4:**
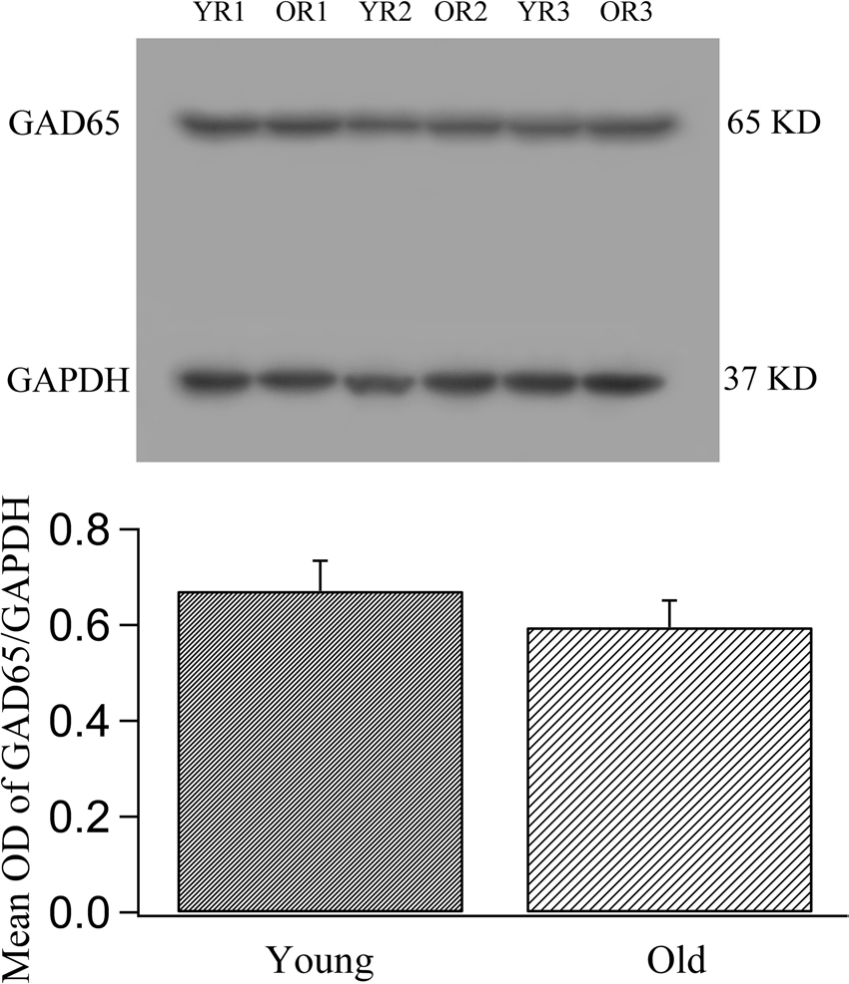
Comparison of the expression levels of the GABA-synthesizing enzyme isoform GAD65 in young adult and old rats. The top panel shows typical GAD65 western blotting results from young rats (YR1, YR2, YR3) and old rats (OR1, OR2, OR3) using GAD65 and GAPDH (glyceraldehyde-3-phosphate dehydrogenase) antibodies (see also the full-length blots presented in Supplementary Figure 1). The bottom panel shows the mean optical density (OD) of GAD65 bands normalized to the OD of the GAPDH band in the two groups of rats. The mean normalized OD value of the GAD65 band in old rats (0.595 ± 0.056) was significantly smaller than that of the GAD65 band in young rats (0.671 ± 0.063) when compared using the one-tailed t-test (p = 0.026), but the difference was marginal when the data were compared using the two-tailed t-test (p = 0.051).

As shown by the above analysis, ageing did not result in significant cell loss in the rat V1 cortical area, but it was associated with a significant decrease in the ratio of GABA-positive neurons to total cortical neurons, indicating that GABA synthesis might be affected by ageing. To explore this possibility, we examined the expression of two GABA-synthesizing enzymes, GAD65 and GAD67. Double labelling of GAD65 and GABA showed that fluorescence indicative of GAD65 immunoreactivity was detectable within all cortical layers in the V1 region of both old and young adult rats. However, GAD65 fluorescence was observed mostly around non-GABAergic neurons and was quite weak in the cell bodies of GABAergic neurons (Fig. 3b,c,e,f,h,i,k, and l), indicating that the GAD65 protein is located primarily within the axons or at the axonal terminals of GABAergic neurons. Although the mean intensity of GAD65 fluorescence in the V1 cortex of old rats (0.136 ± 0.048) was lower than that of young adult rats (0.153 ± 0.045) (one-tailed t-test, p = 0.047), the statistical significance of the difference was marginal (two-tailed t-test, p = 0.0501). In western blots, the mean optical density of the immunoreactive band corresponding to GAD65 protein normalized to the internal reference protein GAPDH was also smaller in old rats (0.595 ± 0.056) than in young adult rats (0.671 ± 0.063) (one-tailed t-test, p = 0.026), but the statistical significance of the difference was marginal (two-tailed t-test, p = 0.051) (Fig. 4, Supplementary Figure 1).

**Figure 5:**
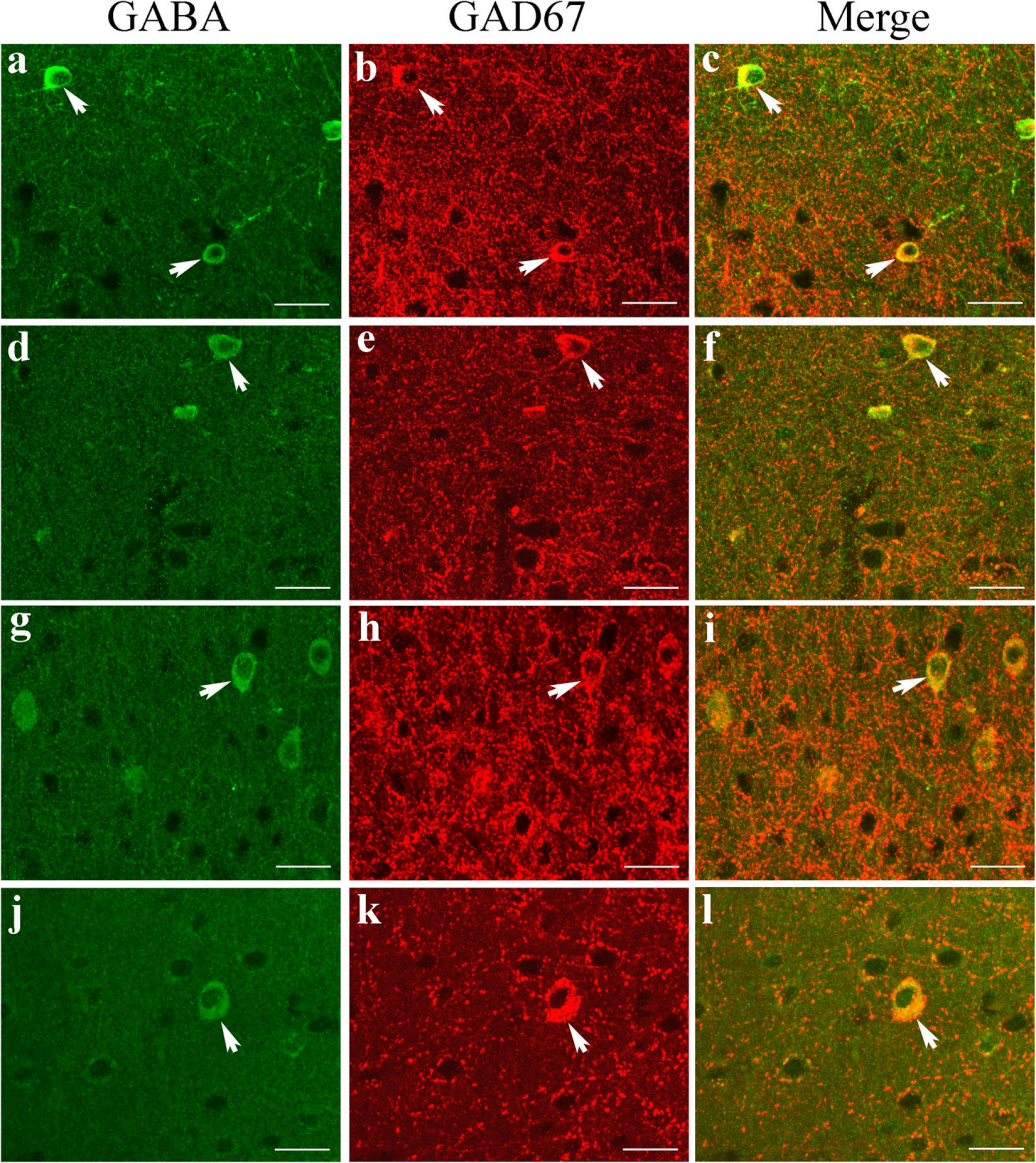
Fluorescence double labelling of GABA and the GABA-synthesizing enzyme GAD67 in different cortical layers in the V1 cortex of young adult (**a**–**c**,**g**–**i**) and old rats (**d**–**f**,**j**–**l**). **a**–**f** and **g**–**l** represent double-labelled samples from cortical layers I–III and IV–VI, respectively. Arrows  indicate typical neurons with strong double labelling of GABA and GAD67. The scale bar equals 20 μm.

**Figure 6:**
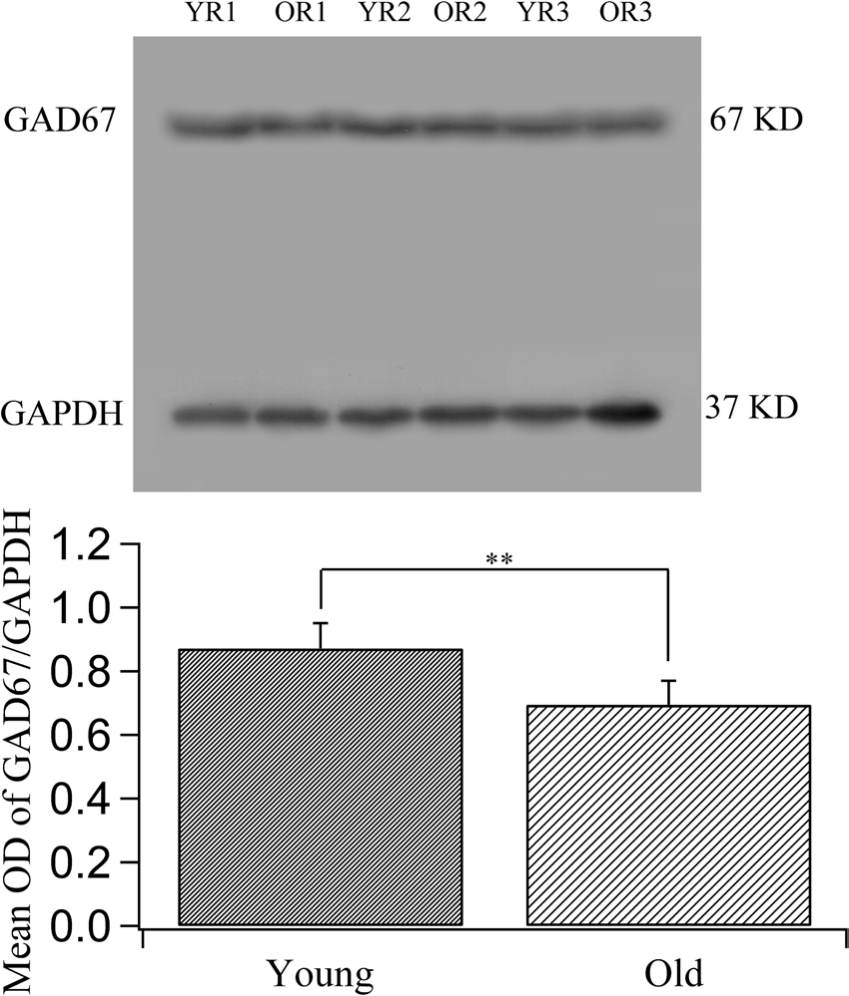
Comparison of the expression levels of the GABA-synthesizing enzyme isoform GAD67 in young adult and old rats. The top panel shows typical GAD67 western blotting results for young rats (YR1, YR2, YR3) and old rats (OR1, OR2, OR3) using GAD67 and GAPDH antibodies (see also the full-length blots presented in Supplementary Figure 2). The bottom panel shows the mean optical density (OD) of the GAD67 band normalized to the OD of the band corresponding to GAPDH in the two groups of rats. The mean normalized OD value of the GAD67 band in old rats (0.694 ± 0.075) was significantly lower than that in young rats (0.872 ± 0.079) (two-tailed t-test, p = 0.0027).

GAD67 immunoreactivity was observed in all cortical layers of area V1 in both old and young adult rats. In double-labelled sections, GAD67 fluorescence was not only strong in the cell bodies of almost all GABAergic neurons but also quite evident around non-GABAergic neurons (Fig. 5b,c,e,f,h,i,k, and l), indicating that the GAD67 protein is distributed both in the cell bodies and the axonal terminals of GABAergic neurons. The distribution of GAD67 protein in the cell bodies of GABAergic neurons was much more marked than that of GAD65. The mean intensity of GAD67 fluorescence in the V1 region of old rats (0.086 ± 0.032) was significantly lower than that in young adult rats (0.166 ± 0.039) (two-tailed t-test, p < 0.000001). Furthermore, western blot analysis showed that the mean optical density ratio of GAD67/GAPDH immunoreactivity in old rats (0.694 ± 0.075) was also significantly lower than that in young adult rats (0.872 ± 0.079) (two-tailed t-test, p = 0.0027) (Fig. 6 and Supplementary Figure 2).

The foregoing analyses demonstrate that ageing significantly reduces the expression of the key GABA-synthesizing enzyme GAD67 in area V1 of the rat cerebral cortex, whereas the expression of GAD65 is only slightly affected by ageing.

### Expression of GABA_A_ receptors

Because previous studies have demonstrated the involvement of ionotropic GABA_A_ receptors in area V1 in the ageing process^5,24^, we examined the expression of the important GABA_A_ receptor subunit α_1_ (GABA_A_R α_1_) in this study.

**Figure 7:**
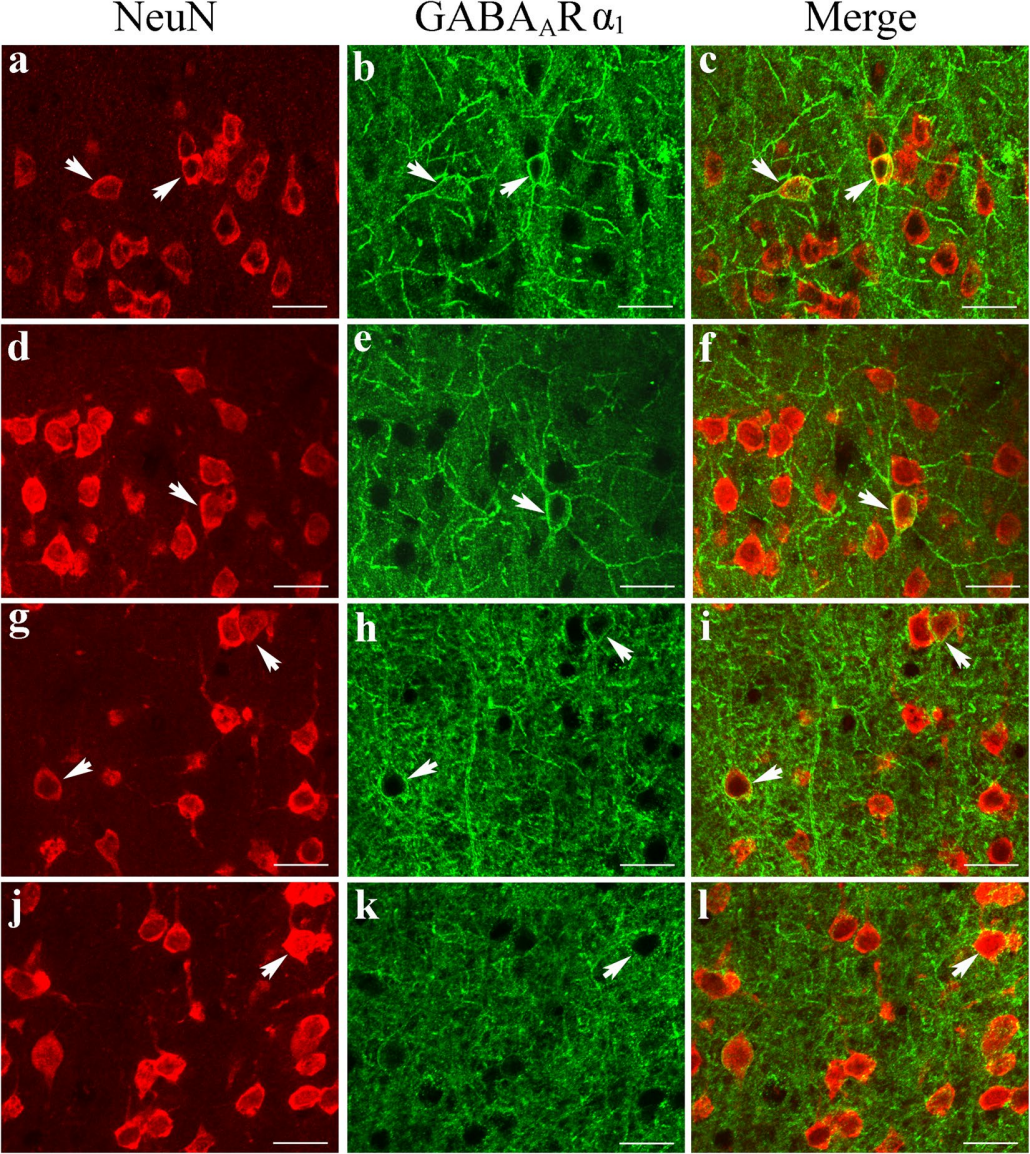
Fluorescence double labelling of cortical neurons (NeuN-positive) and GABA_A_R α_1_ in different cortical layers in the V1 cortex of young adult (**a**–**c**,**g**–**i**) and old rats (**d**–**f**,**j**–**l**). **a-f** and **g**–**l** represent double-labelled samples from cortical layers I–III and IV–VI, respectively. Arrows  indicate typical neurons with GABA_A_R α_1_ fluorescence in the cell body and in axonal/dendritic branches. The scale bar equals 20 μm.

**Figure 8:**
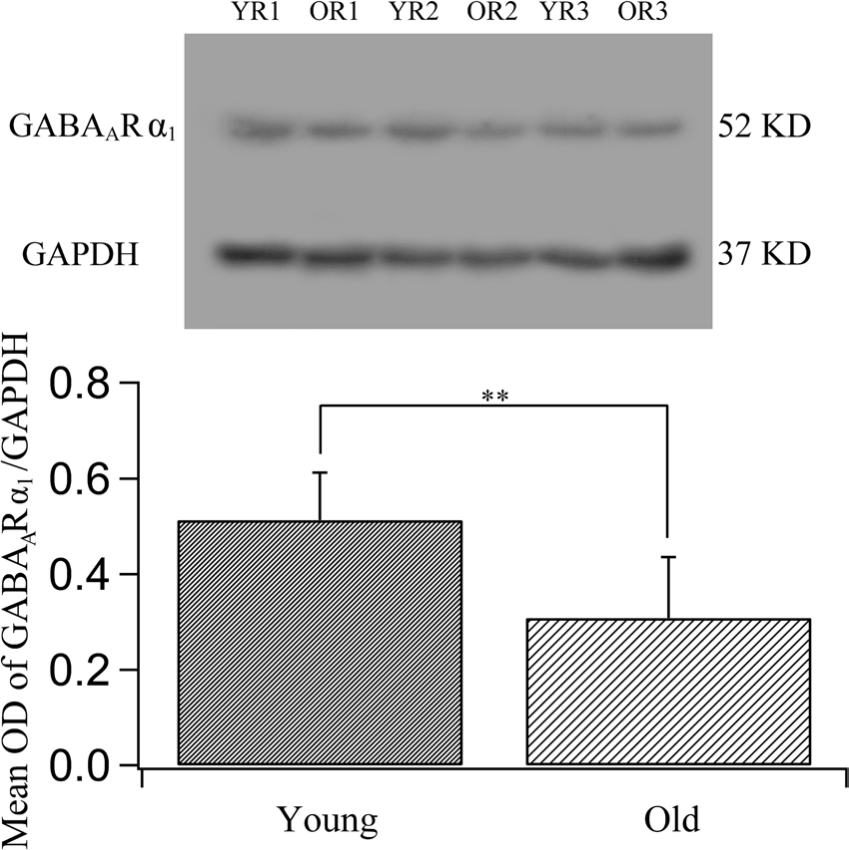
Comparison of the expression levels of the GABA_A_ receptor subunit α_1_ (GABA_A_R α_1_) in young adult and old rats. The top panel shows typical western blotting results obtained for young rats (YR1, YR2, YR3) and old rats (OR1, OR2, OR3) using GABA_A_R α_1_ and GAPDH antibodies (see also the full-length blots presented in Supplementary Figure 3). The bottom panel shows the mean optical density (OD) of the band corresponding to GABA_A_R α_1_ normalized to the OD of the band corresponding to GAPDH in the two age groups. The mean normalized OD value of GABA_A_R α_1_ in old rats (0.308 ± 0.127) was significantly smaller than that in young rats (0.513 ± 0.099) (two-tailed t-test, p = 0.011).

Double-labelling immunofluorescence showed that GABA_A_R α_1_ fluorescence appeared along the dendritic/axonal arbors and around the cell bodies of NeuN-labelled cortical neurons and that it was distributed across all cortical layers in both young adult and old rats (Fig. 7). However, the GABA_A_R α_1_ fluorescence in all cortical layers was weaker in old rats than in young adult rats (Fig. 7). The mean intensity of GABA_A_R α_1_ fluorescence in the V1 cortical region of old rats (0.118 ± 0.035) was significantly smaller than that in the V1 cortical region of young adult rats (0.152 ± 0.037) (two-tailed t-test, p = 0.0000011). Furthermore, western blot analysis showed that the ratio of the optical density of the immunoreactive bands corresponding to GABA_A_R α_1_ and GAPDH was also significantly lower in old rats (0.308 ± 0.127) than in young adult rats (0.513 ± 0.099) (two-tailed t-test, p = 0.011) (Fig. 8 and Supplementary Figure 3). These results show that the expression of GABA_A_R α_1_ in area V1 is significantly attenuated during ageing.

## Discussion

### Age-dependent functional degradation of V1 neurons in rats

This study provides the first evidence that V1 neurons in old rats show significantly decreased response selectivity based on stimulus orientation and direction of motion compared with V1 neurons in young adult rats. This difference was not a result of sampling bias in the V1 region of young adult and old rats. First, among the cells studied in the old and young adult rats, the proportions of simple and complex cells were not significantly different. Furthermore, the proportions of neurons with various orientation (68.4% of cells with OSI ≥ 0.65, 26.5% of cells with 0.4 < OSI < 0.65, and 5.1% of cells with OSI ≤ 0.4) and motion direction biases (38% of cells with DSI ≥ 0.6, 22.8% of cells with 0.4 < DSI < 0.6, and 39.2% of cells with DSI ≤ 0.4) observed in young adult rats in this study were basically consistent with those reported in a previous study of Lister Hooded rats^25^. The mean OSI (0.71 ± 0.18) of the studied neurons of young adult rats in this study was also similar to the value reported recently in normal Long-Evans rats^23^. In addition, the difference in neuronal response selectivity between old and young adult rats observed in this study was not due to variations in the cortical layers in which the recorded neurons of old and young adult rats were located because neurons were randomly sampled at the same depth from the pial surface in both groups of animals, and the proportion of neurons recorded at the depths corresponding to different cortical layers (I, II–III, IV, V and VI)^23^ showed no significant difference between the two age groups.

A systematic analysis of neuronal response indicated that the decreased response selectivity of V1 neurons in old rats was caused by an elevation of intracortical excitation, as indicated by an increased visually evoked response to all visual stimuli, a general increase in spontaneous activity and a decreased signal-to-noise ratio. Our results are consistent with previous findings in old primates and cats^4,6^. Thus, age-related degradation of neuronal function in the visual cortex occurs similarly in different mammalian species.

### Age-associated changes in GABAergic markers in the V1 cortical region of rats

Our data provide new evidence that the proportion of GABA-positive neurons in the V1 cortical region of old rats is significantly lower than that of young adult rats, consistent with previous observations^13,14^. This age-associated difference was not caused by an unequal visualization of GABAergic neurons in the V1 cortical region of old and young rats because we used the same optimized antibody dilution for both age groups. Further, immunohistochemical analysis of sections from young and old rats were performed in batches at the same time, and images were obtained as quickly as possible to avoid fluorescence quenching. In addition, the observed age-related reduction in the proportion of GABA-positive neurons was not caused by biased cell counting, as shown by the following. First, the ratio of GABA-positive neurons to total neurons was calculated based on cell counts performed in series of slices after double immunofluorescent labelling, thus effectively avoiding the over- or underestimation of cell ratios that can occur when cell counts are performed in neighbouring slices after single labelling of GABAergic neurons and Nissl staining of total neurons^13,14^. Second, cell counting was performed by investigators who were blinded with respect to the age groups to which the samples belonged and was performed using the Batch_Process function of Image-Pro Plus^26–29^ with the same parameters for both age groups.

Our statistical analysis showed that the decreased ratio of GABA-positive neurons to total cortical neurons in the V1 region of old rats relative to young rats resulted from a reduction in the number of GABA-positive neurons. The density of total cortical neurons remained stable during ageing. This implies that there may be a loss of GABAergic neurons and a simultaneous proliferation of non-GABAergic neurons in the V1 cortical region of old rats; alternatively, no neuronal loss may occur with age, but some GABAergic neurons in old rats may have been undetectable due to their low GABA levels or may have been transformed into non-GABAergic neurons through reprogramming. Although an increase in the number of neurons in the neocortex at early stages of the postnatal period has been reported^30^, *in vivo* proliferation of neurons in the adult mammalian cortex has not been confirmed except in a few ancient brain regions such as the olfactory system and the hippocampus^31,32^. Cell identity reprogramming has also been observed in the adult cortex, but it occurred only after experimental manipulation of gene expression^33^. Additionally, we carefully checked NeuN-labelled neurons (Fig. 2) in all the brain sections examined in this study and found no evidence of neuronal division in either age group. Therefore, the observed age-related decrease in the number of GABA-positive neurons in this study is likely to have resulted from decreased synthesis of GABA. Consistent with this speculation, our statistical analysis showed that the expression of the key GABA-synthesizing enzyme GAD67, which is distributed primarily in the soma of GABAergic neurons, was significantly decreased in the V1 cortical region of old rats relative to young adult rats, although the expression of GAD65, another GABA-synthesizing enzyme, in old rats was reduced to a level that was only marginally different from that found in young rats.

The effect of GABAergic inhibition depends not only on the GABA level but also on the presence of GABA receptors. Previous studies have shown that ionotropic GABA_A_ receptors play a critical role in the response selectivity of visual cortical neurons^24,34^ and that these receptors may be involved in the degradation of neuronal function during senescence^5^. Therefore, in this study we assessed the expression of an important GABA_A_ receptor subunit α_1_ (GABA_A_R α_1_) in the V1 region of old and young adult rats. Our immunofluorescent labelling and western blotting results consistently showed that the expression of GABA_A_R α_1_ was significantly decreased in old rats relative to young rats, similar to previous findings in the auditory systems of old rats and mice^19,35^.

Taken together, our results indicate that compromised GABAergic inhibition may occur in the rat V1 cortical area during the ageing process. Nevertheless, several factors were not addressed in the current study. For instance, we did not examine age-related changes in other GABA_A_ receptor subunits such as α_2–5_, β_1–3_, γ_1–4_, ρ_1–3_ and δ; these subunits may interact with one another and impact the function of GABA_A_ receptors^19,22^. In addition, other types of GABA receptors such as metabotropic GABA_B_ receptors may also mediate age-associated plasticity in brain function by regulating GABAergic transmission^36^. Further studies are needed to elucidate these issues.

### Mechanisms of age-related decline in neuronal function

A wide range of neuronal function degradation has been reported to occur in the primary and higher-order visual cortical areas of senescent animals. This degradation of function includes weakened response selectivity to visual stimulus orientation and motion direction, decreased response sensitivity to low stimulus contrast, retarded response timing and decreased ability to distinguish visual signals from noise^4,6–11,37–39^. Although these changes in neuronal function could account for the reduction in visual ability that is observed in aged individuals^1–3,40,41^, the cellular and molecular mechanisms that mediate these changes in neuronal function have remained elusive until now^15,42^.

Because neurons in the visual cortex of aged animals show significantly increased excitation, such as a large increase in spontaneous activity and in visually driven responses to all stimuli relative to comparable neurons in young animals, it has been widely suggested that reduced intracortical inhibition may mediate the observed degradation of neuronal function^4,6,15^. Specifically, compromised GABAergic inhibition could be an important underlying factor because modifying GABAergic inhibition can significantly affect neuronal response selectivity in the adult visual cortex^24,43^, and increasing GABAergic inhibition can largely offset declining neuronal function in the aged visual cortex^5^.

To further explore whether reduced GABAergic inhibition occurs during senescence, some researchers have recently examined GABAergic markers in the cerebral cortices of old and young adult subjects using immunolabelling approaches. However, the results reported by different research groups are diverse or even controversial. For example, some studies report a decreased proportion of GABA neurons during ageing^13,14^ whereas others report only the loss of specific GABA neurons^44,45^. With respect to GABA-synthesizing enzymes, some authors suggest that reduced expression of both GAD65 and GAD67 occurs during senescence^17^, whereas others report decreased expression of only GAD65 or GAD67^16,18,22^. With respect to the ionotropic GABA_A_ receptor (GABA_A_R), some authors note a downregulation of different GABA_A_R subunits in the cortex of aged individuals^18–21,46^, others report a compensatory upregulation of GABA_A_R subunits^22^, and still others highlight an age-dependent change in GABA_B_ receptors during senescence^36,47^. Therefore, it is still unclear how GABAergic markers are affected by age and, more importantly, whether the changes reported by different authors are related to the degradation of neuronal function observed in physiological experiments^4,6–11,39^. In this study, we examined and compared neuronal function and the presence of GABAergic markers in the V1 cortical region of old and young adult rats. Our results showed that the response function of V1 cortical neurons declined significantly in old rats relative to young adult rats. At the same time, we found that the V1 cortical layer in aged rats showed a significantly decreased ratio of GABA-positive neurons to total neurons as well as attenuated expression of the key GABA-synthesizing enzyme GAD67 and of the important GABA_A_ receptor subunit α_1_. These results provide direct evidence that compromised GABAergic inhibition may occur in the V1 cortex of old rats and that this compromised GABAergic inhibition accompanies neuronal function decline; thus, it is likely to play a role in mediating the ageing process.

The reasons for the discrepancies in the results concerning the age-related plasticity of GABAergic markers are unclear. It is likely that the GABAergic mechanisms that mediate brain ageing show subtle variations in different mammalian species due to genetic or evolutionary differences. For example, the GABA-synthesizing enzyme GAD65 is preferentially affected by age in primates^18,22^, whereas both GAD65 and GAD67, especially GAD67, change with age in rodents^16,17^, as was observed in the current study. Likewise, different subunits of GABA_A_ receptors, such as the α_1–5_ subunits, also appear to be unequally impacted by age in primates^18,22^ and rodents^19,21^. Another possibility is that aged subjects, even those of the same age, may vary considerably in cortical function due to individual differences in heredity, gender and experience^26,48–50^ and thus may exhibit multiple forms of GABAergic plasticity. These forms of GABAergic plasticity may include a local reduction of GABA synthesis at axonal terminals and compensatory elevation of some GABAergic markers^18,22^, large-scale decrease in the levels of all GABA-synthesizing enzymes, decrease in the number of GABA-positive neurons^13,14,16,17^ and/or downregulation of most GABA receptors^19^. Further studies are required to clarify these possibilities in a way that allows us to determine the neuronal and molecular mechanisms that mediate brain senescence as well as discover anti-ageing solutions for longevity.

## Methods

### Animal preparation

Six young adult rats (male, 3 months old, body weight 200–300 g) and six old rats (male, 22 months old, body weight 500–600 g) were studied in this research. The rats were purchased from the Nanjing Qing-Long-Shan Animal Breeding Farm (Certificate No. SX1207) and reared in the same room at an environmental temperature of 18–25 °C under a 12-h light-dark cycle with food and water *ad libitum*. The animals were examined ophthalmoscopically prior to experimentation to confirm that no optical or retinal problems impaired their visual function. All animal treatments and experimental details were performed in accordance with the guidelines for the Care and Use of Laboratory Animals of the U.S. National Institutes of Health and were approved by the Academic and Ethics Committee of Anhui Normal University.

### Electrophysiological recording and data analysis

The acute *in vivo* single-unit recording procedures used in this study were similar to those used in previous studies^7,23,51,52^. In brief, rats were anaesthetized adequately with urethane (20%, 1.2 g/kg body weight, i.p.) followed by a subcutaneous injection of 0.1 cc of atropine sulphate (0.5 mg/ml). After the paw-withdrawal reflex disappeared, rats were intubated with a plastic tracheal cannula and placed in a modified stereotaxic apparatus. A glucose (5%)-saline (0.9%) solution containing urethane (20 mg/hr/kg body weight) and gallamine triethiodide (10 mg/hr/kg body weight) was administered (0.5–1 ml/hr) by intraperitoneal injection using a syringe pump to keep the animal anaesthetized and paralysed. Artificial respiration was performed, and the expired pCO_2_ was maintained at approximately 3.8%. Body temperature was maintained at 37.5 °C using a heating pad. Heart rate and blood oxygen saturation (SpO_2_ ≥ 94%) were monitored throughout the experiment to assess the level of anaesthesia and to ensure that the animals were in a normal healthy condition. A craniotomy was performed over the primary visual cortex (7–8 mm posterior to bregma and 3–4 mm lateral to the midline) of the left hemisphere with the aid of a surgical microscope (RWD Life Science Inc., Shenzhen, China). The exposed cortical surface was covered with 2% agar to prevent it from drying and to reduce the brain pulsation caused by breathing. Both of the animal’s eyes were kept open using adjustable metal rings surrounding the external portion of the eye bulb, and the optic disk locations were projected onto a tangent screen to determine the vertical meridian. Atropine (0.5%) eye drops were applied to keep the eyes moist throughout the experiment. After the preparations for the experiment were completed, single-unit recordings were performed using a glass microelectrode with an impedance of 3–5 MΩ; the electrode was advanced using a hydraulic micromanipulator (Narishige, Japan). At each penetration site, we isolated units located at approximately the same depth (0–1500 μm) from the pial surface. The neurons from which we recorded were essentially randomly sampled from all cortical layers.

The visual stimuli used in the experiments were moving sinusoidal gratings generated in MATLAB with the aid of extensions provided by the high-level Psychophysics Toolbox^53^ and the low-level Video Toolbox^54^ and presented on a CRT monitor (screen size 35 × 26 cm, resolution 800 × 600, refresh rate 75 Hz) positioned 30 cm from the rat’s eyes. Once a cell’s visually evoked response was detected, the cell’s receptive field centre was preliminarily determined using bars of light emitted from a hand pantoscope and then precisely mapped by presenting a series of computer-generated flashing bars of light on the CRT. We selected optimal spatial frequency (0.01–0.4 cycles per degree), temporal frequency (0.5–16 Hz) and stimulus size for each cell. Each stimulus was presented to the dominant eye. Then, a set of grating stimuli with optimal stimulus parameters, moving in 24 different directions (0–360° scale with an increment of 15°) was used to compile the orientation and motion direction tuning curves. The orientation of each drifting stimulus was orthogonal to its direction of motion. Each stimulus was presented 4–6 times. Prior to each stimulus presentation, the spontaneous activity was obtained while mean luminance was shown on the CRT for 1 s. The duration of each stimulus presentation was less than 5 s, and a 2-min interval was used between stimuli to allow the cell’s functional recovery. The contrast for each stimulus was set at 100%. The mean luminance of the display was 19 cd/m^2^, and the environmental luminance on the cornea was 0.1 lx.

The action potentials of recorded cells were amplified with a microelectrode amplifier (Nihon Kohden, Japan) and a differential amplifier (Dagan 2400 A, USA) and then fed into a window discriminator with an audio monitor. The original voltage traces were digitized using an acquisition board (National Instruments, USA) controlled by IGOR software (WaveMetrics, USA) and saved for on- or off-line analysis. A cell’s response to a grating stimulus was defined as the mean firing rate (spontaneous activity subtracted) corresponding to the time of stimulus presentation, which was used to acquire the orientation tuning curves. Simple and complex cells were classified by computing the modulation ratio of the first Fourier component to the mean component (F_1_/F_0_) of the cell’s visually evoked response; cells with a modulation ratio greater than 1 were considered simple.

**Figure 9:**
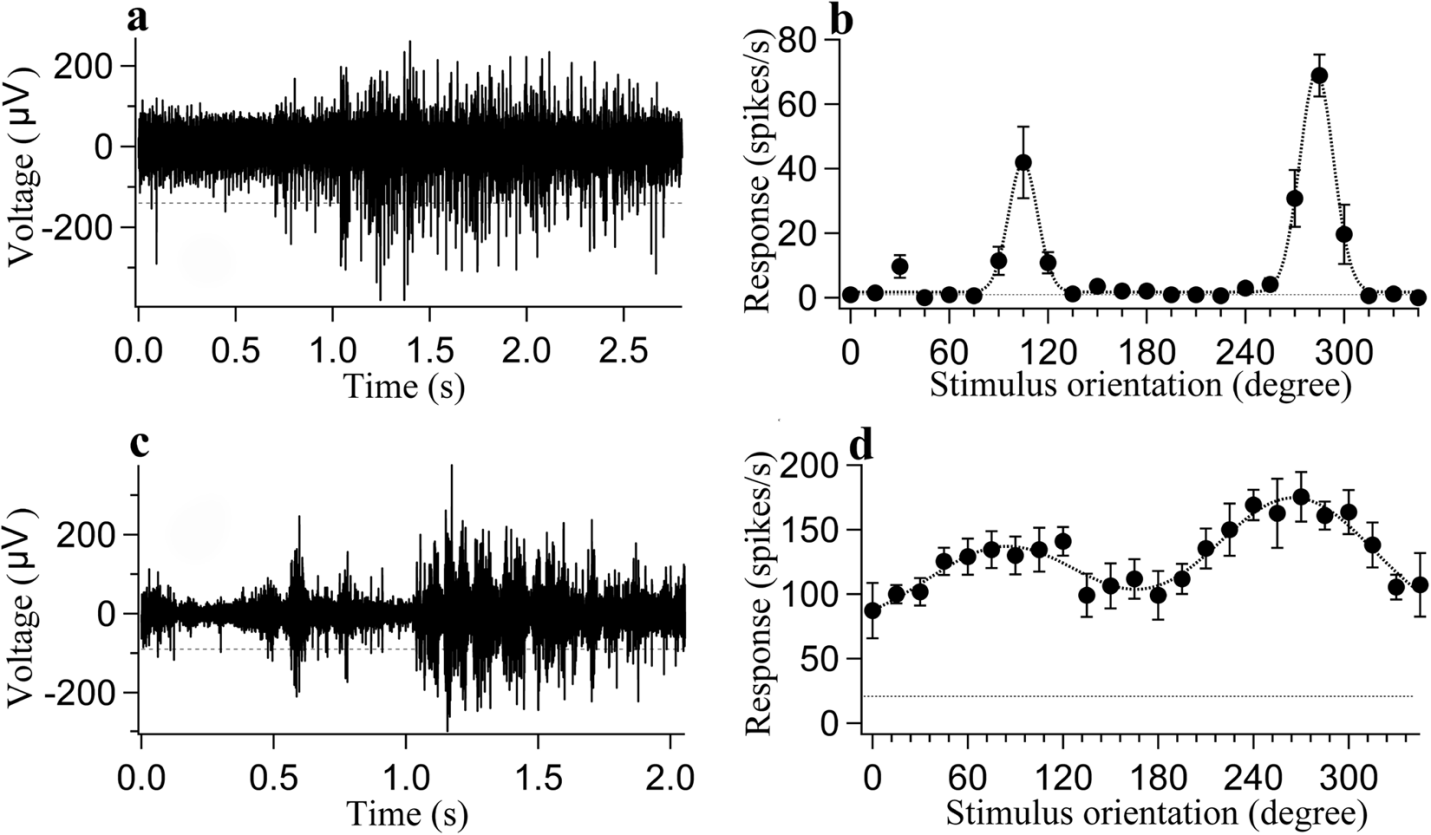
Two sample neurons from young adult (**a**,**b**) and old (**c**,**d**) rats showing the voltage trace, the mean response  as a function of stimulus orientation (0–360 degree) and the curve fitting  of the orientation-tuning curve with a double Gaussian function (**b,d**). The dotted line  at the bottom of each orientation-tuning curve indicates the mean spontaneous activity. (**a,c**) Voltage traces in sample neurons from young (**a**) and old (**c**) rats showing the neuron’s response to its optimal stimulus orientation and motion direction. A spike with an amplitude above the horizontal broken line  was counted as an action potential. Spontaneous activity was acquired during a 1-s pre-stimulus period. The neuron’s visually driven response was evoked by 5 cycles of a drifting grating stimulus presented with optimal stimulus orientation and motion direction. (**b,d**) Orientation-tuning curves of sample neurons from young adult rats (**b**) (fitting error 0.0141, OSI 0.999 and DSI 0.414) and from old rats (**d**) (fitting error 0.0866, OSI 0.438 and DSI 0.243).

To quantify the orientation tuning curve, we fit the firing rate as a function of the orientation and motion direction to a double Gaussian function^55,56^:

1 $$R(\theta )={a}_{0}+{a}_{1}\times exp[\frac{-{(\theta -{\theta }_{0})}^{2}}{2{\sigma }^{2}}]+{a}_{2}\times exp[\frac{-{(\theta -{\theta }_{1})}^{2}}{2{\sigma }^{2}}]$$

where R(θ) is the averaged response to a grating stimulus with motion direction θ, a_0_ is the mean response of the four lowest points in the curve, a_1_ and a_2_ are the amplitudes of the two Gaussians, θ_0_ and θ_1_ indicate the preferred direction and the null direction, respectively, and σ is the standard deviation of the Gaussian function. The goodness of fit of the orientation tuning curve was assessed by computing the mean squared error, and only cells with a fitting error of less than 0.5 were included in the analysis (Fig. 9)^55^.

To make it possible to compare our results to the results obtained in previous studies, we used the following function to quantify the orientation selectivity index (OSI) and motion direction selectivity index (DSI)^23,25^:

2 $$[OSI]\,or\,[DSI]=\frac{{R}_{opt}-{R}_{null}}{{R}_{opt}-{R}_{spon}}$$

where R_opt_ is the response to the optimal orientation and motion direction, R_null_ is the response to the orientation orthogonal to the optimal orientation or the motion direction opposite to the optimal motion direction, and R_spon_ is the spontaneous activity. The larger are the OSI and DSI, the stronger are the orientation and the motion direction selectivity. A cell’s signal-to-noise ratio (STN) was defined as the ratio between the cell’s peak response to the optimal stimulus and the cell’s spontaneous activity. To avoid data skewing or overestimation, all spontaneous activity below 1 spike/s was set equal to 1 spike/s for the signal-to-noise ratio calculation. All of the mean values are expressed as the mean ± standard deviation. Statistical comparisons between young and old rats were conducted using Student’s t-test or two-way analysis of variance (ANOVA).

### Brain tissue preparation

At the end of electrophysiological recording, the brain area containing the V1 region of one hemisphere was completely exposed. After the rat was deeply anaesthetized by an intraperitoneal injection of urethane (20%, 1.8 g/kg body weight, i.p.), the exposed unilateral V1 cortex was quickly removed, frozen in liquid nitrogen, and stored at −70 °C until use in western blot analysis. Immediately after removal of the exposed brain tissue, the rat was transcardially perfused with 0.9% saline followed by 2% paraformaldehyde in 0.1 M phosphate buffered saline (PBS). Then, the cerebral cortex containing area V1 was removed from the skull, post-fixed overnight in 2% paraformaldehyde at 4 °C, and cryoprotected by sequential incubation in 10% (2 h), 20% (2 h) and 30% (overnight) sucrose until tissue sinking. The brain tissue was embedded in OCT compound (Tissue-Tek^®^), and coronal sections were cut at a thickness of 30 µm using a Leica cryostat (Leica Biosystems Inc., Buffalo Grove, IL, USA). Serial frozen sections were collected in order, placed in wells filled with cryoprotectant solution (ethylene glycol-based; 30% ethylene glycol, 30% sucrose, 1% PVP-40, in 0.1 M Phosphate buffer pH 7.4) and temporarily stored at −20 °C for subsequent immunofluorescent labelling.

### Fluorescence immunohistochemistry

Fluorescence immunohistochemical reactions were performed on free-floating sections using methods similar to those described previously^17,57,58^. First, the sections were pre-incubated with QuickBlock™ Blocking Buffer (Beyotime, P0260) for 1 h. After washing in PBS (10 min × 3), the sections were incubated overnight at 4 °C with primary antibodies diluted in QuickBlock™ Primary Antibody Dilution Buffer (Beyotime, P0262). An optimal dilution was predetermined to visualize target neurons in the V1 cortical region of both age groups of rats. The primary antibodies used in this study included: rabbit anti-GABA (1:300, Sigma, A2052), mouse anti-NeuN (1:1000, Abcam, ab104224), mouse anti-GAD65 (1:500, Sigma, AMAB91048), mouse anti-GAD67 (1:6000, Sigma, G5419) and rabbit anti-GABA_A_R α_1_ (1:200, Abcam, ab33299). After several washes in PBS, the sections were incubated with secondary antibodies in QuickBlock™ Secondary Antibody Dilution Buffer (Beyotime, P0265) for 2 h at room temperature. The secondary antibodies included anti-Mouse IgG H&L Alexa Fluor^®^ 555 (1:1000, Abcam, ab150114) and anti-Rabbit IgG H&L Alexa Fluor^®^ 488 (1:1000, Abcam, ab150077). After secondary antibody incubation and several washes in PBS, the sections were mounted on gelatin-coated glass slides, coverslipped with glycerol and sealed with nail polish. Adjacent control sections were labelled simultaneously using the same procedure as described above with the exception that the primary antibody was replaced by PBS.

After immunofluorescent labelling, images were obtained as soon as possible using a confocal laser scanning microscope (FV1000, Olympus) with a 20× or 60× objective. Automated sequential acquisition of multiple channels was used. The frame size was either 1024 × 1024 pixels or 512 × 512 pixels. For each image, ten confocal planes were Z-stacked with a step of 0.56 µm. Stacks of images were merged into a maximum intensity projection and saved in tiff format. Image analysis was performed using Image-Pro Plus 6.0 software (MediaCybernetics, Bethesda, USA)^26–29^ by experimenters who were blinded to the age groups of the animals from which the images were obtained. Images from 10 slices from each animal were sampled randomly for data analysis. The number of total cortical neurons (NeuN-positive neurons) and the number of GABA-positive neurons in each cortical layer (layers I, II–III, IV, V and VI) in each sample were counted within the same AOI (area of interest) using the Batch_Process function of Image-Pro Plus after spatial calibration, area filtration and colour segmentation (HIS). AOIs were traced and sampled according to the cortical layer identification of NeuN-labelled neurons. The mean density of total cortical neurons and GABA-positive neurons, as well as the proportion of GABA-positive neurons in each cortical layer, were calculated based on the cell count across multiple AOIs. The mean fluorescence intensity of GAD65/67 and GABA_A_R α_1_ in each slice was measured across 30 randomly selected AOIs using a customized macro plugged into the Batch_Process script of Image-Pro Plus after calibration of background intensity and segmentation of target colour. The mean values in young adult and old rats are expressed as the mean ± standard deviation.

### Western blotting

Western blotting was performed as described previously^18,26,59,60^. In brief, frozen V1 tissues were cut, weighed, thawed, and homogenized in 10 volumes of an ice-cold buffer (25 mM Tris–HCl pH 7.6, 150 mM NaCl, 1% NP-40, 1% sodium deoxycholate and 0.1% SDS) and a protease inhibitor cocktail (Kang Chen Biotechnology, Shanghai, China) and centrifuged at 12,000 rpm for 15 min at 4 °C. Protein concentration in the supernatant was measured using Coomassie Brilliant Blue G-250 (Sangon Biotechnology, Shanghai, China). Proteins (50 µg) in cortical samples obtained from individual rats were fractionated using 8% sodium dodecyl sulphate polyacrylamide gel electrophoresis (SDS-PAGE) and transferred onto polyvinylidene fluoride (PVDF) membranes (Beyotime Biotechnology, Shanghai, China). The membranes were blocked with 5% nonfat dry milk in Tris-buffered saline (TBS)-Tween 20 for one hour and incubated overnight at 4 °C in TBS-Tween 20 containing primary antibodies, including rabbit anti-GAD65 (1:200; Abcam, ab26113), rabbit anti-GAD67 (1:500, Abcam, ab97739), rabbit anti-GABA_A_R α_1_ (1:200, Abcam, ab33299) and rabbit anti-GAPDH (glyceraldehyde-3-phosphate dehydrogenase) (1:5,000; Sangon Biotechnology). The membranes were washed 3 times for 5 min in TBS-Tween 20 and incubated with peroxidase-conjugated AffiniPure Goat Anti-Rabbit IgG (1:5,000, AB10058, Sangon Biotechnology, China) diluted in TBS-Tween 20 for 2 h at 25 °C and washed again in TBS-Tween 20. The membranes were then developed with BeyoECL Plus (Beyotime Biotechnology, Shanghai, China), and the signal was visualized on Kodak X-OMAT LS film (Sigma). The optical density (OD) of bands on western blots was measured using Image-Pro Plus software. The OD values of the immunoreactive bands corresponding to GAD65, GAD67 and GABA_A_R α_1_ are expressed relative to the OD values of the GAPDH bands in each sample.

### Data Availability Statement

The datasets generated during and/or analysed during the current study are included in this article and are also available from the corresponding author on request.

## Electronic supplementary material

Supplementary Figures
