# Supplementary material for: Changes in GABAergic markers accompany degradation of neuronal function in the primary visual cortex of senescent rats
Source: Sci Rep. 2017 Nov 2;7:14897. doi: 10.1038/s41598-017-15006-3 (PMC5668371; doi:10.1038/s41598-017-15006-3)
Supplement: Supplementary file 1 — Supplementary Figures [file 41598_2017_15006_MOESM1_ESM.pdf]

Changes in GABAergic markers accompany degradation of neuronal function in the primary visual cortex of senescent rats

Yanxia Ding[1+], Yuan Zheng[1+], Tao Liu[1+], Ting Chen[1], Changhua Wang[1], Qiushuang Sun[1], Mutian Hua[2], Tianmiao Hua[1*]

Supplementary Figures and Legends

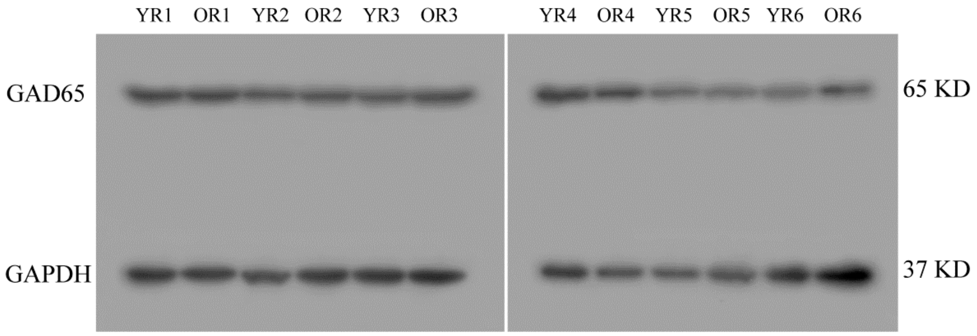

Supplementary Figure 1. Western blots of the primary visual cortex (V1) of young rats (YR1-6) and old rats (OR1-6) using GAD65 and glyceraldehyde-3-phosphate dehydrogenase (GAPDH) antibodies. All samples were derived from the same experiment, and the blots were processed in parallel.

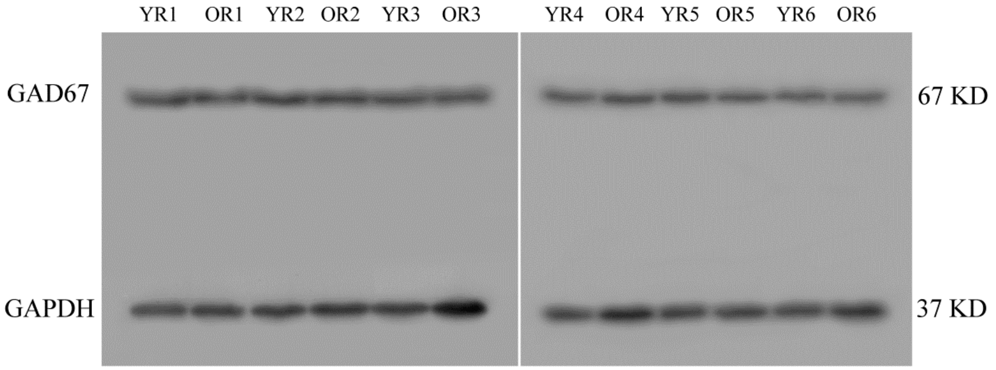

Supplementary Figure 2. Western blots of the primary visual cortex (V1) of young

rats (YR1-6) and old rats (OR1-6) using GAD67 and GAPDH antibodies. All samples were derived from the same experiment, and the blots were processed in parallel.

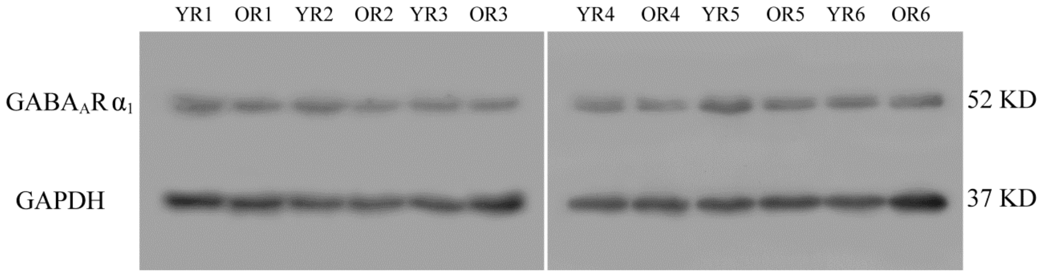

Supplementary Figure 3. Western blots of the primary visual cortex (V1) of young rats (YR1-6) and old rats (OR1-6) using $GABA_AR$ α_1 and GAPDH antibodies. All samples were derived from the same experiment, and the blots were processed in parallel.
